# Chemotactic and Inflammatory Responses in the Liver and Brain Are Associated with Pathogenesis of Rift Valley Fever Virus Infection in the Mouse

**DOI:** 10.1371/journal.pntd.0001529

**Published:** 2012-02-28

**Authors:** Kimberly K. Gray, Melissa N. Worthy, Terry L. Juelich, Stacy L. Agar, Allison Poussard, Dan Ragland, Alexander N. Freiberg, Michael R. Holbrook

**Affiliations:** 1 Department of Microbiology and Immunology, The University of Texas Medical Branch, Galveston, Texas, United States of America; 2 Department of Pathology, The University of Texas Medical Branch, Galveston, Texas, United States of America; 3 Galveston National Laboratory, The University of Texas Medical Branch, Galveston, Texas, United States of America; 4 Integrated Research Facility, National Institute of Allergy and Infectious Diseases, National Institutes of Health, Frederick, Maryland, United States of America; 5 Institute for Human Infections and Immunity, The University of Texas Medical Branch, Galveston, Texas, United States of America; 6 Center for Tropical Diseases, The University of Texas Medical Branch, Galveston, Texas, United States of America; University of Texas Medical Branch at Galveston, United States of America

## Abstract

Rift Valley fever virus (RVFV) is a major human and animal pathogen associated with severe disease including hemorrhagic fever or encephalitis. RVFV is endemic to parts of Africa and the Arabian Peninsula, but there is significant concern regarding its introduction into non-endemic regions and the potentially devastating effect to livestock populations with concurrent infections of humans. To date, there is little detailed data directly comparing the host response to infection with wild-type or vaccine strains of RVFV and correlation with viral pathogenesis. Here we characterized clinical and systemic immune responses to infection with wild-type strain ZH501 or IND vaccine strain MP-12 in the C57BL/6 mouse. Animals infected with live-attenuated MP-12 survived productive viral infection with little evidence of clinical disease and minimal cytokine response in evaluated tissues. In contrast, ZH501 infection was lethal, caused depletion of lymphocytes and platelets and elicited a strong, systemic cytokine response which correlated with high virus titers and significant tissue pathology. Lymphopenia and platelet depletion were indicators of disease onset with indications of lymphocyte recovery correlating with increases in G-CSF production. RVFV is hepatotropic and in these studies significant clinical and histological data supported these findings; however, significant evidence of a pro-inflammatory response in the liver was not apparent. Rather, viral infection resulted in a chemokine response indicating infiltration of immunoreactive cells, such as neutrophils, which was supported by histological data. In brains of ZH501 infected mice, a significant chemokine and pro-inflammatory cytokine response was evident, but with little pathology indicating meningoencephalitis. These data suggest that RVFV pathogenesis in mice is associated with a loss of liver function due to liver necrosis and hepatitis yet the long-term course of disease for those that might survive the initial hepatitis is neurologic in nature which is supported by observations of human disease and the BALB/c mouse model.

## Author Summary

Rift Valley fever virus (RVFV) is a deadly virus that is found primarily in sub-Saharan Africa. However, mosquitoes that are capable of transmitting RVFV have a worldwide distribution. RVF affects a broad range of animal species and is known to cause encephalitis or hemorrhagic fever in humans with survivors having long-term health effects such as a loss of vision. There is no vaccine currently approved for use in humans. In this study we evaluated the host response to infection with either a RVFV vaccine strain or a wild-type strain to identify similarities or differences that could be exploited for development of therapeutics or improved vaccines. While mice infected with the vaccine strain did not develop disease and survived, infection with the wild-type strain caused severe disease with a fatal outcome. Analyzing multiple clinical factors and the host immune response over the course of infection allowed us to identify potential host factors associated with disease progression during wild-type virus infection. This work also provides support for a current vaccine candidate, MP-12, in demonstrating a limited and protective host response to infection.

## Introduction

Rift Valley fever virus (RVFV) (family *Bunyaviridae*, genus *Phlebovirus*) is a highly pathogenic virus that can cause lethal disease in both humans and ruminant animals. RVFV is classified by the National Institute for Allergy and Infectious Diseases (NIAID) as a category A priority pathogen and is a United States Department of Agriculture (USDA) high priority pathogen. RVFV is historically endemic in sub-Saharan Africa, but recent outbreaks on the Arabian Peninsula have indicated an increased range for this virus. A recent outbreak in South Africa (2010–May 2011) resulted in over 250 laboratory confirmed cases with an approximate case fatality rate of 11% [1]. RVFV is transmitted primarily by *Aedes mcintoshi* mosquitoes, although the virus has been detected in 23 species of mosquitoes [2]. Given the abundance of *Aedes* spp. in the United States and in other parts of the world, the potential introduction of RVFV into naïve populations is a very serious agricultural and public health concern [3], [4]. Effective vaccines or therapeutic intervention are not commercially available for prevention or treatment of Rift Valley fever (RVF) in humans.

RVF outbreaks in human populations vary in size, intensity and location with these parameters dependent upon rainfall and mosquito abundance [5]–[7]. The largest recorded outbreak of RVF was in Egypt in 1977 with 10,000 to 20,000 human cases [8], [9]. Patients present with general malaise (chills, headaches, joint pain and nausea) and fever that is debilitating for a short period. A small percentage (≤1%) of patients present with severe manifestations including encephalitis, retinal vasculitis, or hemorrhagic fever. Mortality rates up to 20% have been reported in these cases [9]–[12].

RVFV causes a much more catastrophic disease in ruminants than is seen in humans, particularly in pregnant animals where nearly 100% of RVFV infected ruminants abort their fetuses [13], [14]. The onset of human cases can often be predicted by outbreaks of disease in animals [7], [15].

Although there are currently no licensed vaccines or therapeutics for RVFV infection in humans, several potential vaccines are available and have been tested in animals and humans (www.clinicaltrials.gov) [16]–[22]. One of the most promising is the live-attenuated MP-12 vaccine [18] which was developed by the US Army [23]. The MP-12 strain was developed by 12 serial passages of the wild-type strain ZH548 in the presence of 5-fluorouracil to generate a virus that was avirulent in pregnant ewes [22], [23]. Further analysis of MP-12 found that it induced high titer neutralizing antibodies in vaccinated ewes and protected vaccinated lambs from subsequent challenge with wild-type virus [24]. MP-12 has subsequently been found to be apathogenic in adult mice [25]. The MP-12 vaccine is available to researchers through the Special Immunizations Program at the US Army Medical Research Institute of Infectious Diseases (USAMRIID) as part of an on-going clinical trial (www.clinicaltrials.gov).

RVFV is a lipid-enveloped icosohedral virus [26]–[28] containing a segmented, negative-sense, single-stranded RNA genome [29]. The genome consists of three segments: large (L), medium (M), and small (S). The L segment encodes the viral polymerase, while the M segment encodes 4 proteins: Gn and Gc, (surface glycoproteins), NSm (non-structural protein) and a 78 kDa protein. The S segment is ambisense and encodes for two proteins: N (nucleocapsid) and NSs (non-structural protein) [29].

The NSs protein of wild-type virus has been shown to have type I interferon (IFN) antagonist activity while this property is lacking in the NSs protein of the RVFV mutant clone 13 which has an in-frame deletion in the NSs gene [30], [31]. Other than the potential role of type I IFN in limiting disease [32]–[34], little else is known about the host immune response to RVFV infection.

One study by Smith *et al.* examined pathogenesis in BALB/c mice following wild-type virus challenge, focusing on viral titers in tissues, types of infected cells and histopathological changes during infection [35]. An additional recent study evaluated the gene expression of IL-10, IFN-β and IFN-γ over the course of wild-type virus infection in juvenile BALB/c mice while utilizing gene array to identify the significant upregulation of a number of immune response genes in the liver of infected mice [33].

The study described here was developed with the intent of characterizing the relationship between the host response and viral pathogenicity in adult C57BL/6 mice following challenge with either wild-type RVFV ZH501 or the attenuated vaccine strain MP-12. Here we evaluated a large panel of cytokines and chemokines in multiple tissues over the complete course of the infection in addition to measuring a number of clinical parameters. The ZH501 strain of RVFV was isolated during the same 1977 outbreak in Kenya as was ZH548 and a sequence comparison indicates approximately 99% identity. We chose to use C57BL/6 mice for this study as this strain is biased toward a Th-1, or cell-mediated, immune response, which is more typical in limiting viral infection [36]–[42].

Our studies found a marked difference between the host response to infection with wild-type RVFV and the attenuated MP-12 vaccine strain. While neither virus caused overt changes in body weight or temperature, changes in clinical parameters were more profound in animals infected with wild-type virus than with the vaccine strain. Histopathologic changes in animals infected with the wild-type virus were more significant than those in mock or MP-12 infected animals, including hepatocellular necrosis, which correlated with the presence of viral antigen. Furthermore, there was a significant cytokine response in wild-type virus infected animals in all organs examined; whereas the only changes seen in MP-12 infected animals were, surprisingly, in the brain. An increased cytokine response could also be correlated with histological changes seen in the organs of ZH501 infected animals versus those infected with MP-12. These studies reinforce the notion that the pathogenicity seen in RVFV infections in mice is driven primarily by an unregulated host inflammatory response which results in significant loss of liver function and development of neurologic disease.

## Methods

### Ethics statement

This animal study was conducted in accordance with an animal use protocol approved by the Institutional Animal Care and Use Committee (IACUC) (Protocol #0505029) at the University of Texas Medical Branch (UTMB) following recommendations in the Guide for the Care and Use of Laboratory Animals of the National Institutes of Health. This institution also accepts as mandatory the PHS policy on Humane Care of Vertebrate Animals used in testing, research and training. All work was performed at UTMB in a facility accredited by the American Association for the Accreditation of laboratory Animal Care.

### Cell lines and viruses

RVFV wild-type strain ZH501 and vaccine strain MP-12 were obtained from Dr. John Morrill (UTMB). The virus stocks used for the assays described here were p2 (MP-12) and p1 (ZH501) from the stocks received from Dr. Morrill. Both viruses were cultured and titrated on Vero E6 cells (ATCC # CRL-1586). For virus challenge of mice, virus was diluted in serum-free DMEM prior to inoculation.

### Mouse challenge studies

Adult (8–10 week old), female C57BL/6 mice (Harlan Sprague Dawley) were inoculated subcutaneously with 1,000 plaque forming units (PFU) of ZH501, MP-12 or an equivalent volume of diluent. The LD_50_ for ZH501 is ∼1 PFU. Each challenge group consisted of 5 animals. Three challenge groups (one group each: ZH501, MP-12 and mock infected) were terminally bled and euthanized every 12 hours post-infection (hpi). Mice from groups designated for sacrifice on the last day of the experiment had their weight and body temperature measured daily throughout the course of the experiment by BioMedic Data Solutions (Seaford, DE) transponders implanted two days prior to challenge. All work involving handling of infectious RVFV ZH501, was performed in the Robert E. Shope BSL-4 laboratory at UTMB.

At sacrifice, whole blood was collected for hematology and clinical chemistry and serum was isolated for analysis of the cytokine response profile and for virus titration. Approximately half of each organ we examined (liver, spleen and brain) was harvested and homogenized in 0.5 ml PBS using a Qiagen TissueLyser (Qiagen) to be used for virus titration and cytokine profile analysis. All samples removed from BSL-4 laboratory were γ-irradiated (5 Mrad) prior to analysis.

### Virus titration

Vero E6 cells were infected with 100 µl of sample through 10-fold serial dilutions. Plates were incubated for 1 hour in a 37°C incubator with 5% CO_2_ and gentle rocking every 15 minutes. A 0.8% tragacanth (f/c)/1× MEM overlay was applied to the wells. After 3 days, the overlay was removed and the cells were stained with 0.2% crystal violet diluted in 10% formalin. The plaques were counted and the titers determined. Viral titers are reported as log_10_ PFU.

### Bio-plex assay

Tissue homogenates and serum were processed according to manufacturer instructions and then analyzed using a Bio-plex 200 system (Bio-Rad, Hercules, CA). Briefly, the samples were centrifuged for 10 minutes at 1,000 rpm at 4°C to remove debris. The supernatants were collected and aliquoted into 96-well plates in pre-determined wells; this plate was centrifuged at 1,250 rpm to remove any remaining debris. The supernatant was transferred to a 96-well flat bottom plate and processed for use on the Bio-plex system. The cytokines were coupled to cytokine specific multi-plex beads (Bio-Rad) following the manufacturer's instructions using pre-designed assays that measured the concentration of a panel of cytokines including interleukin (IL)-1α/β, IL-2, IL-3, IL-4, IL-5, IL-6, IL-9, IL-10, IL-12 (p40, p70), IL-13, IL-17, eotaxin, IFNγ, KC (CXCL1), monocyte chemoattractant protein (MCP)-1 (CCL2), granulocyte colony-stimulating factor (G-CSF), granulocyte macrophage colony-stimulating factor (GM-CSF), macrophage inflammatory protein (MIP)1α/β (CCL3/CCL4), tumor necrosis factor (TNF)-α and RANTES (CCL5).

### ELISA

Assays for IFN-β were performed by ELISA following the manufacturer's instructions (PBL Biomedical Laboratories, Piscataway, NJ). Samples were prepared similarly to the Bio-plex samples. They were centrifuged for 10 minutes at 1,000 rpm at 4°C to remove debris. The supernatant was transferred to ELISA strips processed for use on the plate reader. The IFN-β in the sample adhered to the pre-coated wells during incubation. After washing, the plates were incubated with HRP-conjugated secondary antibody, with a washing step between incubations. The strips were then incubated with a TMB solution for 15 minutes and 100 µl of stop solution was added. These strips were read at 450 nm within 5 minutes of addition of stop solution.

### Clinical evaluation

Complete blood counts (CBC) were evaluated with a Hemavet hematology analyzer (Drew Scientific, Dallas, TX). Analysis included total white (WBC) and red blood cell (RBC) counts, platelet counts, hemoglobin concentration, hematocrit, and the counts of white blood cell subpopulations (lymphocytes, monocytes, eosinophils, neutrophils, and basophils). Clinical chemistry analysis was performed using a VetScan2 Chemistry Analyzer (Abaxis, Union City, CA). Fourteen analytes were examined including the liver enzyme alanine aminotransferase (ALT), serum glucose, amylase, plasma electrolytes (calcium, phosphorus, sodium, and potassium), globulin, albumin, total bilirubin, urea nitrogen, creatinine, and total protein.

### Immunohistochemistry (IHC)

Formalin-fixed tissues were processed in a Sakura Tissue-Tek VIP Processor, and were mounted in paraffin blocks. The RVFV antigen immunostain was prepared using mouse on mouse polymer technology and the Sequenza cover plate system. The steps briefly were: 1) Antigen retrieved using DIVA solution in a steamer (20 min); 2) Peroxide block using peroxidase (5 Min); 3) Blocking step using Rodent Block M (30 min); 4) RVFV mouse hyper-immune ascitic fluid (World Reference Collection for Emerging Viruses and Arboviruses at UTMB), used at 1∶100 dilution (60 min); 6) Secondary antibody using Biocare MM HRP Polymer (30 min); 7) chromagen was DAB (5 min); 8) Counterstained with hematoxylin for 5 minutes.

### Statistical analysis

As the test groups are small, a Fisher's exact test was used to determine the significance between ZH501, MP-12 and mock infected groups in clinical and cytokine analyses. A Kaplan-Meier estimate weight and temperature analysis was applied to the 6 days post infection (dpi) group.

## Results

### Clinical observations

**Figure 1:**
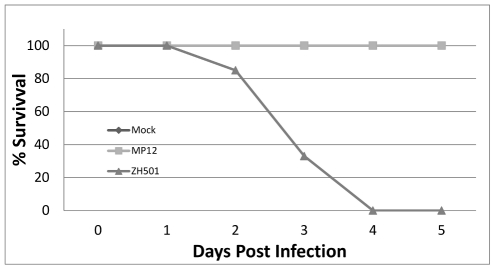
Mouse survival after infection. Percent survival of mice that were infected with vaccine strain MP-12, wild-type strain ZH501 or were mock infected. Animals included in this figure were from a predetermined subset of animals that were analyzed for temperature and weight changes (Figure 2). Of all animals included in this study, only three ZH501 infected animals survived to 96 hpi. All mock and MP-12 infected animals survived until euthanized for tissue collection.

**Figure 2:**
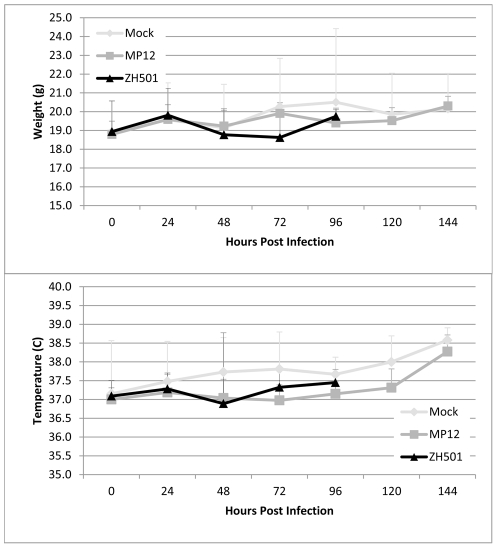
Mouse daily weight and temperature. Daily weight (g) and temperature (°C) of mock-infected C57BL/6 mice and mice infected with either MP-12 or ZH501.

As anticipated, mock and MP-12 infected mice showed no signs of clinical disease and all survived until sacrificed or the end of the experiment. ZH501 infected mice began to show signs of illness (i.e. ruffled fur and hunched posture) by 36 hpi. All ZH501 infected mice developed signs of clinical illness and succumbed to disease. Of the subset of ZH501 infected animals monitored daily for weight and body temperature, 85% were alive on day 2, 30% survived to day 3, and all were dead at day 4 (Figure 1). All animals in the monitored MP-12 and mock-infected groups survived through completion of the study. During the course of the experiment, none of the mice lost a significant amount of weight or became febrile (Figure 2).

### Complete Blood Counts (CBCs)

**Figure 3:**
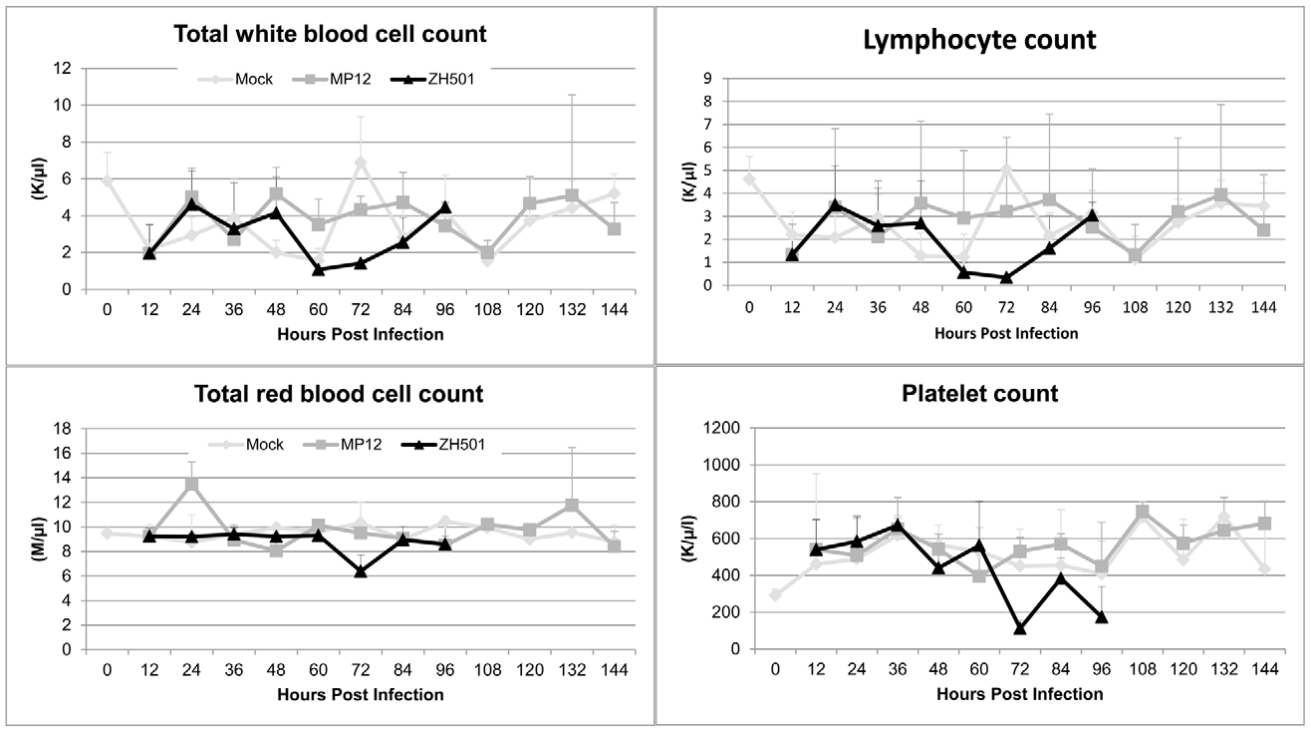
Hematology results. Total white blood cell, lymphocyte, red blood cell and platelet counts over the course of disease in mock (◊), MP12 (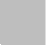) or ZH501 (▴) infected animals. Each data point represents the mean of 5 animals per group except at 96 hpi in the ZH501 group where only 3 animals remained.

CBCs are an important diagnostic and prognostic tool for determining overall human clinical health and are a reliable measure of multiple indices of whole blood components. We performed CBCs in this study to determine their utility in assessing the health of mice during RVFV infections. Whole blood from animals was collected by terminal cardiac puncture. Overall, no significant difference in the total white blood cell (WBC) count between mock and MP-12 infected mice was observed for any of the measured parameters (Figure 3, Table S1). The CBC's of ZH501 infected animals were not remarkably different from MP-12 or mock infected animals until 60 hpi where drop in total WBC count was observed between 60 and 72 hpi, but rebounded at 84 and 96 hpi. The decrease in total white blood cells between 60 and 72 hpi was driven primarily by marked reduction in lymphocyte and monocyte numbers in the ZH501 infected animals. At 96 hpi the concentration of monocytes in ZH501 infected animals was elevated relative to both mock and MP-12 infected animals, but the difference was not significant. Eosinophils only showed a change at 96 hpi after ZH501 infection when the concentration of eosinophils was increased in ZH501 infected animals. The neutrophil population did not deviate significantly between the infection groups over the course of the experiment (Table S1).

RBC populations were largely the same between all three challenge groups. However, at 24 hpi the RBC concentration in MP-12 infected animals was significantly higher than in mock or ZH501 infected mice while at 72 hpi, ZH501 infected animals had a decreased RBC count (Figure 3, Table S1). Interestingly, the platelet count was within normal ranges (592–2,972 K/ul) for all groups until 72 hpi where the platelet concentration in ZH501 infected mice dropped well below normal. There was a slight recovery at 84 hpi, but then the level dropped to below normal again at 96 hpi.

### Serum chemistry

**Figure 4:**
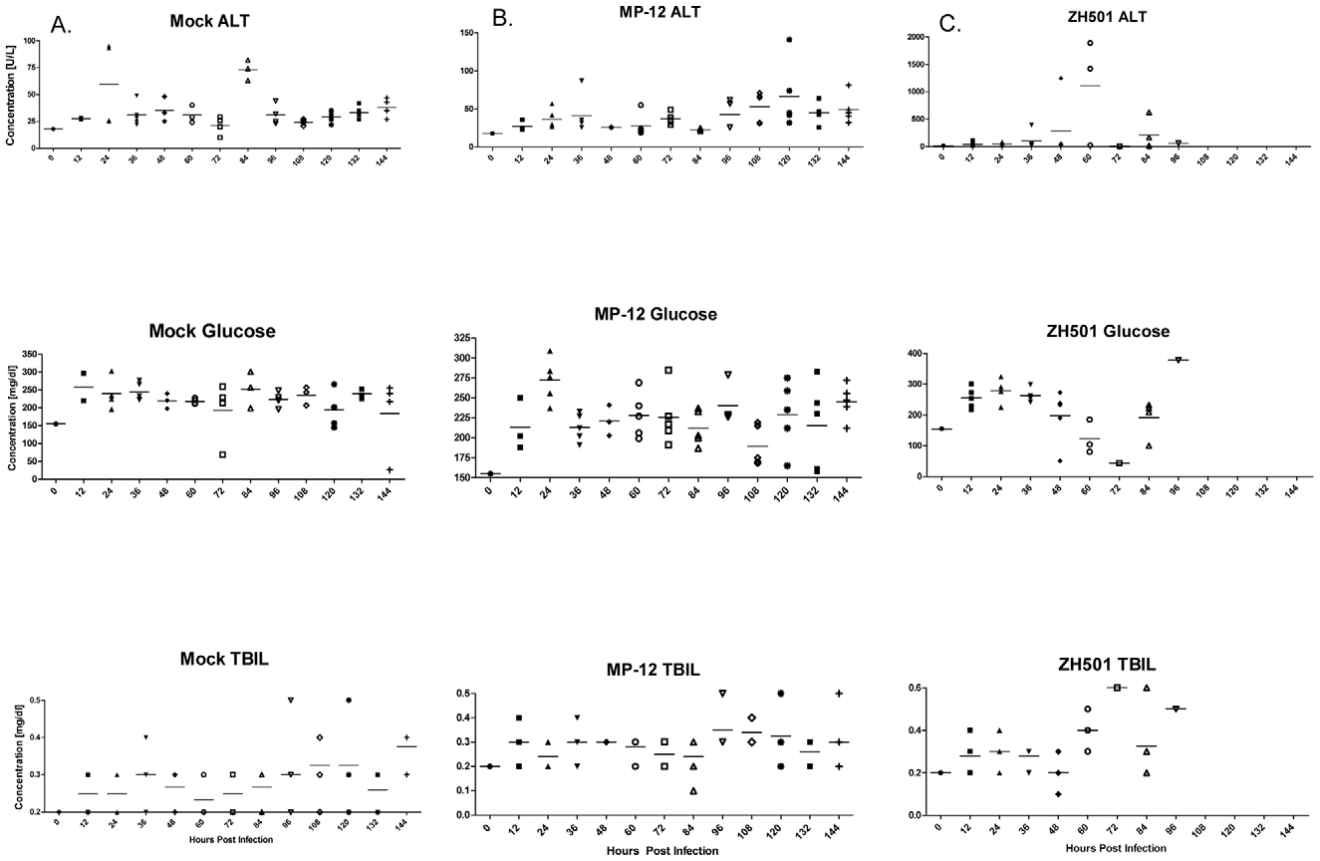
Liver function enzymes. Alanine aminotransferase (ALT), glucose, and total bilirubin concentrations in the serum of mock (A), MP-12 (B) and ZH501 (C) infected mice. The first row provides serum ALT concentrations, the second row glucose concentrations and the third total bilirubin concentrations. Each symbol represents an individual mouse. There were five mice in each group, except at 96 hpi where only three animals had survived until this point of the study. The horizontal bar represents the mean of the mice for that group. Please note that the maximum for the Y-axis in (C)-ALT is 2000 U/L rather than 150 U/L as in (A) and (B) ALT graphs.

RVFV is known to be hepatotropic [43] and infection can stimulate release of liver specific enzymes from disrupted hepatocytes and serve as clinical markers of hepatic dysfunction. In this study serum liver enzyme concentrations became elevated in ZH501 infected mice as the disease progressed. Alanine aminotransferase (ALT) began to increase at 36 hpi and peaked in concentration at 60 hpi in ZH501 infected animals (Figure 4C). While normal values of ALT in the mouse are 17–77 U/L, one mouse infected with ZH501 had ALT serum levels over 500 U/L at 36 hpi, one animal had levels of 1,300 U/L at 48 hpi, and two mice had over 1,500 U/L of ALT in their serum at 60 hpi. No animals in the mock or MP-12 infected groups had ALT concentrations above 150 U/L (Figure 4A–B).

Serum glucose levels dropped drastically in ZH501 infected mice at 72 hpi (Figure 4C), began to increase at 84 hpi, and were above normal at 96 hpi. Total bilirubin was also elevated in ZH501 infected animals (Figure 4C). At 72 and 84 hpi one mouse in each ZH501 infected group had elevated total bilirubin concentrations. Analyzed plasma electrolytes (calcium, phosphorous, sodium, and potassium) did not show a significant deviation from normal concentrations (data not shown).

### Virus distribution

**Figure 5:**
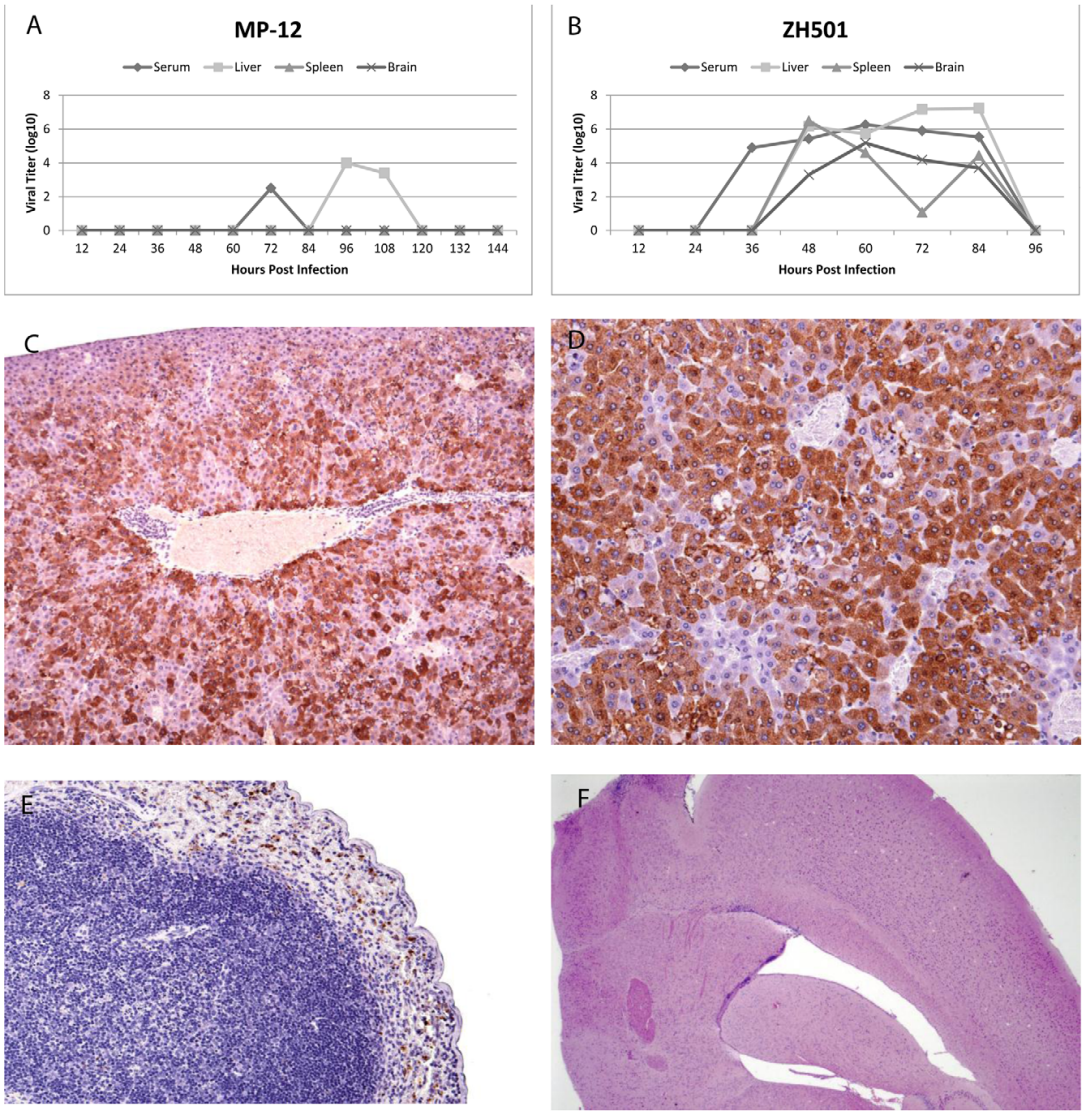
Viral Titers. Viral titers in the serum, liver, spleen, and brain after infection with (A) MP-12 or (B) ZH501. Each point with the exception of 96 hpi in the ZH501 graph represents the mean virus titer of five mice. The 96 hpi points in the ZH501 graph represent only the 3 mice that survived until that point. Error bars for the standard deviation were removed for clarity. (C) Immunohistochemical staining for RVFV antigen in liver from a ZH501 infected mouse at 60 hpi. Intracytoplasmic viral antigen is depicted by brown staining, 10×. (D) Immunohistochemical stain of liver from a ZH501 infected mouse at 84 hpi. Intracytoplasmic viral antigen is depicted by brown staining, 20×. (E) Immunohistochemical stain of spleen for RVFV antigen from a ZH501 infected mouse at 84 hpi. The red pulp sinusoids contain numerous cells with cytoplasmic brown, granular material depicting viral antigen. There are no viral antigen positive cells in the lymphoid follicle. Note the increased lymphocytolysis depicted by pyknotic nuclei and cellular fragments, 20×. (F) Brain from a ZH501 infected animal at 84 hpi with no pathologic changes, H&E 2.5×.

To correlate the onset of illness with virus replication in different organs, we harvested tissues from groups of 5 mice every 12 hours and determined virus titers by plaque assay. MP-12 infected animals did not have any detectable virus in any tissue until they became viremic at 72 hpi with a peak titer of 2.5 log_10_ PFU/ml. The viremia was below detectable limits (∼200 PFU) by 84 hpi in our assay. In the liver, virus appeared at 96 hpi when the viral load was just under 4 log_10_ PFU/ml with a slight decrease in tissue titer at 108 hpi and was no longer detectable at 120 hpi. MP-12 was not detected in the spleens or brains of infected mice at any point during the study (Figure 5A). ZH501 appeared at 24 hpi in the serum and peaked at 84 hpi with over 7 log_10_ PFU/ml in the liver (Figure 5B). In the spleen the peak viral titer was seen at 48 hpi where over 6 log_10_ PFU/ml of virus was present. While there was a fall in the spleen titers at 72 hpi, the viral titers in the serum, liver, and brain remained elevated. ZH501 titers peaked at 60 hpi in the brain and serum at 5 log_10_ PFU/ml and 6 log_10_ PFU/ml, respectively. Virus titers declined to levels undetectable by plaque assay in the serum, liver and brain in the three remaining animals sacrificed at 96 hpi (Figure 5B).

Immunohistochemical stains for RVFV antigen in formalin-fixed tissues collected from the three study groups were examined microscopically (Figures 5C–F). RVFV antigen was not found in the tissues of 10 mock-infected animals analyzed over the course of the study. Seven of 11 MP-12 infected mice had no viral antigen in any of the tissues examined while three mice, one each at 48, 72, and 96 hpi, had occasional antigen-positive cells in splenic red pulp sinusoids and white pulp lymphoid follicles. One MP-12 infected animal at 96 hpi exhibited cytoplasmic antigen staining within multiple hepatic microgranulomas. Seven of eight ZH501 infected mice sacrificed from 48 to 84 hpi exhibited diffuse granular cytoplasmic viral antigen staining in most hepatocytes (75% to 95% of cells affected) (Figures 5C–D). While two of eight ZH501 infected animals had no splenic viral antigen staining, up to 5% of the cells in splenic red pulp sinusoids and white pulp lymphoid follicles in the other six animals contained cytoplasmic viral antigen (Figure 5E). Viral antigen was not detected in brain or spinal cord sections in any mouse from any infection group (Figure 5F).

### Cytokine profiles

RVFV infection is known to cause a number of pathogenic effects that could be correlated with the host immune response to infection. In order to identify potential mechanisms of pathogenesis during RVFV infection, we examined the cytokine response and histological changes in three major organs (spleen, liver, brain) and serum. We chose these organs because they are principal components of the host immune response and have been associated with RVFV pathogenicity [43], [44]. The results from these assays found that wild-type ZH501 causes a significant cytokine response in all of the organs examined compared to MP-12. The responses for each organ are summarized below. Individual graphs for a representative subset of the cytokines examined are shown in Figures S1, S2, S3, S4.

#### Serum (Figure S1)

The serum from RVFV infected animals was evaluated for changes in cytokine levels relative to mock-infected animals. These studies indicated a mild and limited inflammatory response in MP-12 infected animals with slight elevations of IL-6, IL-12 and MIP-1α around 72 hpi. Elevated IL-12(p70) and IFN-γ suggest a Th-1 response against viral infection. In contrast, the response against ZH501 infection was very broad with onset evident at 48 hpi with significant elevations of IL-6, KC, MCP-1 and MIP-1α. By 72 hpi, virtually all cytokines measured in ZH501 infected animals were significantly elevated relative to mock infected animals. The concentrations of IL-6, G-CSF and MCP-1 indicate a strong inflammatory response and potential response to vascular leakage [45]. Interestingly, the majority of these cytokines return to non-significant levels in animals sampled at the 84 hpi time-point although IL-6, IL-8, G-CSF and MCP-1 remain elevated.

#### Liver (Figure S2)

Liver samples from MP-12 infected animals showed no significant deviation from mock-infected control animals with the exception of a decrease of IL-12(p70) at 72 hpi. As with serum samples, liver homogenates from ZH501 infected animals had evidence of an inflammatory response with increases in KC, MCP-1 and MIP-1α. A number of cytokines, such as IL-1α, IL-12, G-CSF and KC, became elevated at 48 hpi with most peaking at 72 hpi. G-CSF and KC concentrations were both very high with peak concentrations several hundred fold higher than mock infected controls. MCP-1 and IL-1α were also elevated although not to the extent of G-CSF and IL-8.

#### Spleen (Figure S3)

The response to MP-12 infection in the spleen was much like that seen in the serum except that cytokine peaks were delayed by approximately 24 hpi. As seen in the serum, IL-6, IL-12(p70) and IFN-γ concentrations peaked at levels that were significantly higher than in mock infected animals. In ZH501 infected animals, cytokine concentrations began to increase significantly beginning at 48 hpi. IL-6 and G-CSF had the largest fold changes, 200 and 450 times higher, respectively, relative to mock. There was no significant change in concentrations of IL-2, IL-4, IL-12(p70) or IFN-γ suggesting that there was little, if any, stimulation of T- or B-cell activation or differentiation in the spleen. However, significant induction of IL-6, IL-1α, and several chemokines, particularly MCP-1, indicates recruitment of pro-inflammatory cells into the spleen. The peak cytokine response in the spleen (72 hpi) correlates with a significant drop in viral titer in the spleen (see Figure 4B).

#### Brain (Figure S4)

The cytokine response in the brain indicates that the brain is involved during infection with both MP-12 and wild-type ZH501. This is particularly interesting given that we did not detect any virus in the brain of MP-12 infected animals by plaque assay (Figure 4A) nor were there any signs of neurologic disease. In MP-12 infected animals, IL-2, IL-13 and IL-17 were all elevated along with MCP-1 and MIP-1α. The presence of IL-2, IL-13 and IL-17 suggests the presence and activation of a B-cell response [46], [47]. MCP-1 and MIP-1α concentrations were elevated soon after infection indicating early onset recruitment of immunomodulatory cells. The brains of ZH501 infected mice showed an early response similar to that seen in MP-12 infected animals, however, concentrations of these cytokines remained elevated and many of their downstream cytokines also became elevated with peak concentrations seen at 60–72 hpi. IL-6 was not evident in the brains of MP-12 infected animals and IL-8 did not deviate significantly from mock infected animals. In ZH501 infected animals both cytokines were elevated significantly indicating a strong pro-inflammatory response. G-CSF levels were also extremely high 48–72 hpi (over 2,500 times higher than mock and MP-12 infected mice) suggesting stimulation of a strong protective anti-apoptotic response in the brain that is also associated with neurogenesis [48]–[50]. Although there was no clear clinical or histological evidence that the ZH501 infected animals developed neurologic disease, the cytokine response strongly suggests that they were going down a path that could lead to encephalitis.

**Figure 6:**
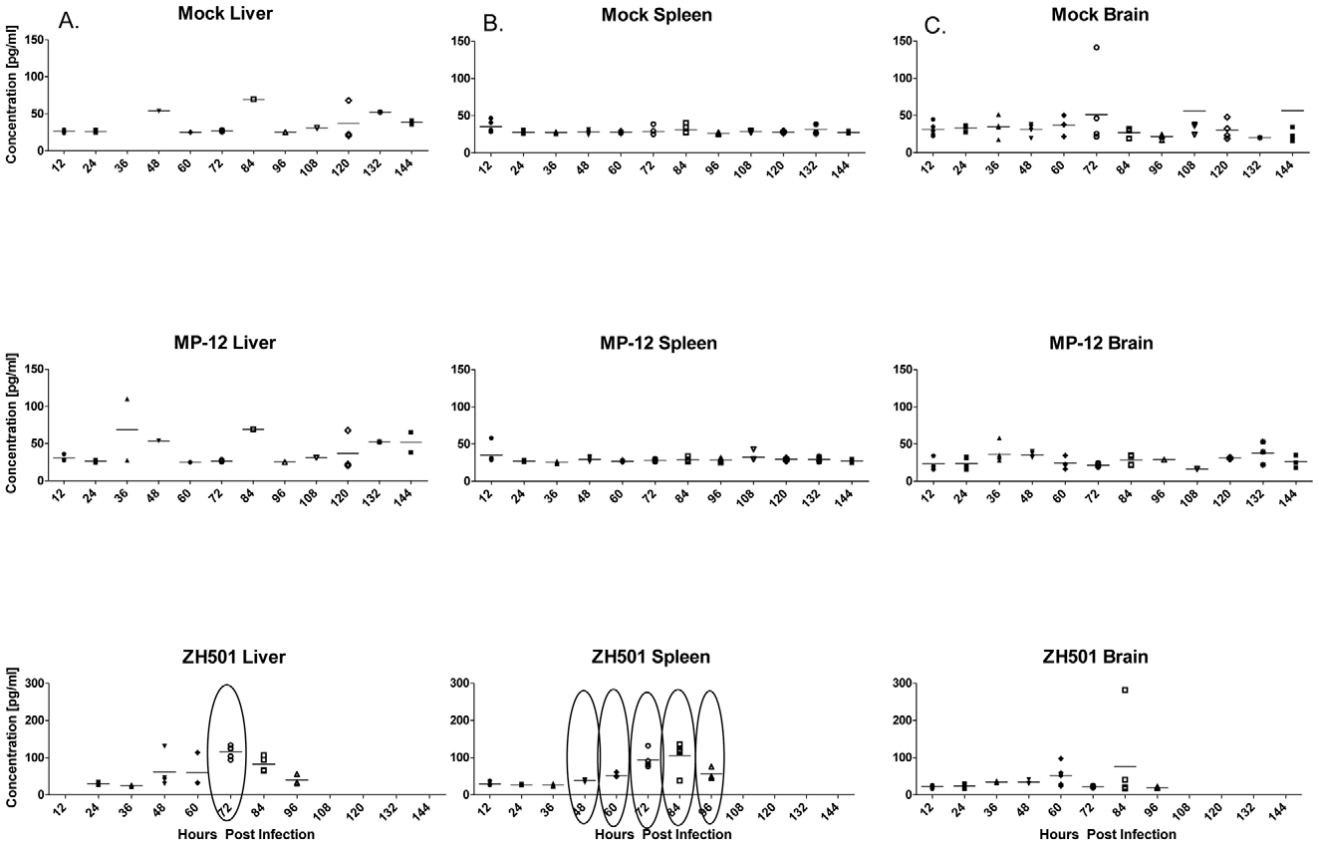
IFN-β concentration following infection. IFN-β concentration in the (A) liver, (B) spleen, and (C) brain in mice after infection. The first row represents mock infected mice, the second row represents MP-12 infected mice, and the third is ZH501 infected mice. Each symbol represents an individual mouse. There were five mice in each group with the exception of the 96 time-point for ZH501 when only three animals were surviving. The horizontal bar represents the mean of the mice for each group. Brackets indicate that the average value for ZH501 infected animals is significantly different from both mock and MP-12 infected animals.

IFN-β (Figure 6)-As wild-type RVFV has been shown to have type I IFN antagonist properties [30], [31], [34], we examined IFN-β production in the liver, spleen and brain. One ZH501 infected animal had elevated IFN-β in the brain at 84 hpi. Three mock infected animals had slightly elevated IFN-β concentrations in the brain while none of the other mock infected animals exceeded 75 pg/ml in any sample tested. Similar results were seen in MP-12 infected animals, but one animal had elevated IFN-β in the liver at 36 hpi. However, ZH501 infected animals had elevated IFN-β in the liver and spleen of the majority of animals tested at 72 and 84 hpi. Mean concentrations of IFN-β in the spleens of ZH501 infected mice were significantly higher (2–5 fold increase) than both mock and MP-12 infected animals between 48–96 hpi, while in the liver a significant difference was only seen at 72 hpi.

### Histopathology

**Figure 7:**
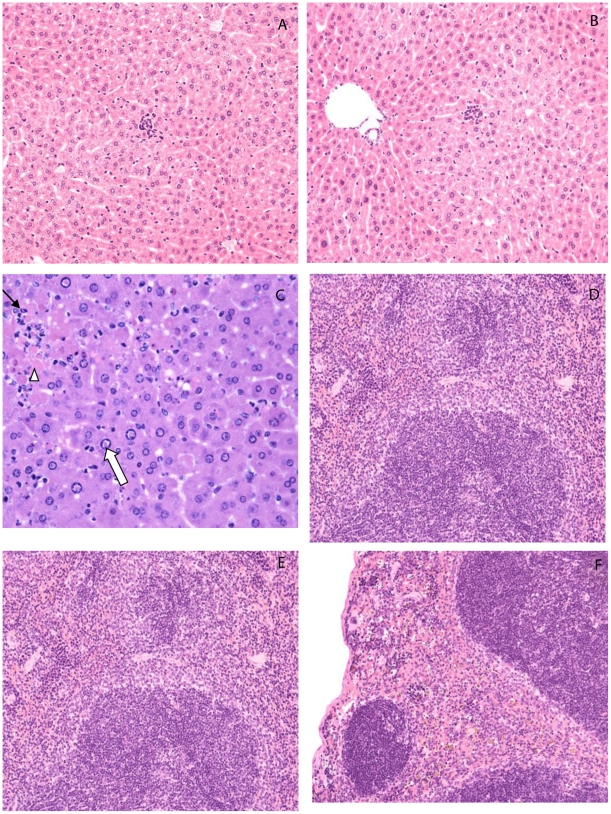
Liver and spleen pathology. (A) Liver from a mock-infected mouse at 84 hpi. The liver is essentially normal H&E 20×. (B) Liver from an MP-12 infected mouse at 84 hpi. The random, small microgranulomas composed of individually-necrotic hepatocytes surrounded by small numbers of neutrophils and mononuclear cells are unrelated to the study, H&E 20×. (C) Liver from a ZH501 infected mouse at 84 hpi, showing piecemeal hepatocyte necrosis (white arrowhead), hepatocellular intranuclear eosinophilic inclusions (white arrow). Note the presence of neutrophils (black arrow) within foci of necrosis, H&E stain 40×. (D) Spleen from a mock-infected mouse at 84 hpi. The spleen is essentially normal, H&E 20×. (E) Spleen from an MP-12 infected mouse at 84 hpi. The spleen is essentially normal, H&E 20×. (F) Spleen from a ZH501 infected mouse at 84 hpi with moderate amounts of necrotic cellular debris and macrophages containing hemosiderin (brown pigment) within red pulp sinusoids, H&E 10×.

Examination of H&E stained sections from the liver, spleen and brain largely supported clinical and cytokine response observations indicating significant damage to the liver and spleen of ZH501 infected animals, while tissues from mock and MP-12 infected animals were essentially normal (Figures 7 A, B, D, E).

Histological evidence of hepatocellular degeneration and necrosis was seen as early as 48 hpi in ZH501 infected mice, and was present throughout the remaining course of the infection. These findings correlated well with the cytokine response in that ZH501 infected animals with the more significant cytokine response also had increased tissue damage. At 48 hpi, the mouse with the highest cytokine levels (IL-1α, IL-6, IL-8, IL-12, IFNγ, eotaxin, and G-CSF) also exhibited moderate diffuse hepatic necrosis. Liver changes in the mouse with lower cytokine levels at 48 hpi were much less severe and consisted of diffuse cytoplasmic vacuolation of hepatocytes and random small microgranulomas. At 60 hpi, the mouse with higher levels of IL-1α, IL-6, IL-8, IFNγ, and various chemokines in the liver had moderate necrosis and hepatocyte degeneration, while other ZH501 infected animals had minimal microgranulomas composed of neutrophils, Kupffer cells, and lymphocytes that did not affect surrounding tissue. The foci of hepatocyte degeneration and necrosis contained occasional neutrophils, Kupffer cells and lymphocytes. At 72 hpi, the livers of ZH501 infected mice had evidence of severe architectural disruption and most of the hepatocytes were in the process of degeneration and necrosis. There was disassociation of hepatocytes from hepatic plates, expansion of sinusoidal lumens and accumulation of blood (hemorrhage) within dilated hepatic sinusoids. Most of the hepatocyte nuclei that remained intact contained a single, centrally-located, intranuclear eosinophilic inclusion that tended to peripheralize the nuclear chromatin. At 84 hpi, the livers looked similar to those collected at 72 hpi with marked architectural disruption and necrosis (Figure 6C).

The spleens of the mock and MP-12 infected mice remained essentially normal throughout the study (Figures 7D, E). The only pathologic changes occurred in mice infected with ZH501. From 48 hpi to 84 hpi, the spleens of ZH501 infected mice with the highest levels of cytokines (IL-1α, IL-1β, IL-6, eotaxin, MCP, and MIP-1α) also had corresponding splenic enlargement and moderate amounts of necrotic cellular debris diffusely within red pulp sinusoids. Similar splenic changes were not seen in animals with a limited cytokine response. At later time points (i.e. 72 and 84 hpi), however, splenic pathology was more profound, consisting of increased neutrophilic and histiocytic infiltrates admixed with hemosiderin and necrotic cellular debris (Figures 7F).

The brains of all mice appeared essentially normal throughout the course of the study despite evidence of a high viral titer and significantly increased inflammatory cytokine concentrations in the brain tissue of some study animals.

## Discussion

Rift Valley fever virus is a highly pathogenic virus that causes large scale outbreaks in livestock with frequently associated epidemics in humans. The mouse is an established model for RVFV infection where animals succumb to wild-type RVFV infection within a matter of days with disease characteristics similar to that seen in fatal cases of human disease and in infected newborn lambs [12]. While the mouse is an accepted model for disease, the host response to infection has not been well characterized. Some studies have evaluated clotting time, mortality, and disease progression, but few have focused in on the host immune response to RVFV infection [12], [22], [32]–[35], [51]–[53]. Smith *et al.* recently described the pathogenesis of wild-type ZH501 infection in BALB/c mice following subcutaneous challenge [35], but did not have the benefit of cytokine profiles to correlate pathogenesis to the host response while other studies focused on a limited number of cytokines [32]–[34].

The study described here is the first broad examination of the host response to RVFV infection in the mouse model. This study identifies very significant differences between the host response to infection with wild-type ZH501 or the live-attenuated vaccine strain MP-12 and clearly demonstrates that a significant and systemic pro-inflammatory immune response is likely a major contributor to the progression to lethal disease.

Unlike previous studies where we have evaluated the clinical response to viral infection in the mouse model [54], clinical analysis of RVFV infection in the mouse provided indicators of disease onset and development that correlated with reports of RVFV infection in both humans and primates. In the present study, there was no statistical difference in body weight or temperature in any of the challenge groups despite the ZH501 infected animals showing clear indications of disease and individual animals in the dpi 3 and 4 ZH501 infected groups losing weight. However, hematological analysis made it clear that wild-type RVFV infection stimulates depletion of lymphocytes, monocytes and platelets over the course of disease. The depletion of total WBC was largely the result of lymphopenia during the mid-stages of disease in ZH501 infected animals. Our study found a decrease in both total WBC and lymphocytes between 48 and 60 hpi in ZH501 infected mice (Table S1) with a gradual recovery toward the end of the study. Lymphocyte recovery may be the result of stimulation of the hematopoietic system as significantly increased concentrations of G-CSF were found in the serum and tissues of ZH501 infected animals. Hematology results in the C57BL/6 mouse model correlate well with previously published studies in BALB/c mice [35] and rhesus macaques [52]. Infected macaques had an initial leukocytosis and then a sharp decrease to leukopenia at 5 dpi that slowly resolved by 7 dpi. In another study evaluating WBC counts in ZH501 infected rhesus macaques, animals with a fatal outcome had evidence of leukocytosis at 2 dpi which remained elevated until death or returned to baseline levels just prior to death [53]. Animals which showed clinical illness but survived infection had only a transient leukocytosis.

The decrease in platelet concentration in ZH501 infected mice was not surprising given the hemorrhagic nature of the disease. Morrill *et al*. reported a drop in platelet counts in RVFV infected macaques which was in direct proportion to the severity of the disease [53]. In our mouse studies there was no difference between MP-12 and mock infected animals, but there was a significant decrease in the mean platelet count in ZH501 infected animals relative to mock infected animals. Examination of individual ZH501 infected animals found that mice with liver damage indicated by elevated serum ALT, low glucose and high bilirubin also had low platelet counts. However, not all mice with low platelet counts had evidence of severe liver damage. These data indicate a correlation between liver disease and low platelet counts, but not the reverse, in mice infected with wild-type RVFV. Similar results have been seen in humans where approximately half of the patients with laboratory confirmed RVF present with abnormal platelet counts [55].

Clinical chemistry data provided a clear indication of liver disease in ZH501 infected animals, but no evidence of similar disease in MP-12 or mock infected animals. Liver necrosis has been shown to be a large contributor to the mortality seen after infection with wild-type RVFV [11], [35], [53], [55]–[57]. In studies with ZH501 infected non-human primates, serum aminotransferases were increased in animals with fatal infections [58]. The same study also showed a correlation between the amount of liver damage (using ALT values) and viremia [58]. ZH501 infected mice with elevated ALT and bilirubin concentrations had histological evidence of hepatitis, liver necrosis and possible hepatitis associated jaundice. In Saudi Arabia, 95% of patients with confirmed RVFV infections had increased ALT levels and 75% resulted in liver failure [55]. Animals with extremely elevated (above 600 U/L) serum ALT also had increased virus titers and a stronger cytokine response in the liver indicating a strong correlation with the onset of liver disease. At necropsy the livers were enlarged, appeared congested, and had areas of black discoloration. Microscopic examination revealed hepatic necrosis, loss of sinusoidal integrity, hemorrhage, and significant levels of viral antigen (Figure 7C).

To characterize the host immune response to RVFV infection and to differentiate infection with MP-12 vaccine strain from wild-type ZH501, we determined the concentration of a panel of cytokines in tissue homogenates and serum. The response to ZH501 infection was significant and dramatic in all of the tissues tested while the response to MP-12 infection was largely unremarkable. The response seen in livers from ZH501 infected animals was unexpected. Given the nature of the wild-type RVFV infection, we expected evidence of a significant inflammatory response in the liver. The induction of a pro-inflammatory response was not evident; however, a significant chemokine response focused on recruitment of T-cells, neutrophils and monocytes was pronounced. Histological evaluation of livers from ZH501 infected mice identified significant cellular infiltration and congestion with the appearance of neutrophils, macrophages, and lymphocytes within disrupted sinusoids and eosinophilic intranuclear inclusions that are reported to be characteristic of RVFV infection [59]. Histological data correlated strongly with the observed chemokine response and were also consistent with studies in BALB/c mice [35].

Morphologically, the spleen was enlarged and the red pulp contained accumulations of cellular debris, neutrophils and macrophages containing hemosiderin (Figure 7C). Hemosiderin is commonly found in macrophages when they break down hemoglobin and release heme and bilirubin after conditions that result in hemorrhage [60], [61]. The presence of hemosiderin containing macrophages in the spleen is indicative of hemorrhage following ZH501 infection.

The level of serum cytokines was significantly increased in ZH501 infected animals. The levels of IL-6, IL-12(p40), G-CSF and several chemokines were elevated indicating the onset of a strong inflammatory response and recruitment of immunomodulatory cells. The concentrations of all analytes, with the exception of IL-5 and IL-13, were significantly elevated in serum at 72 hpi, supporting our hypothesis of a strong unregulated immune response following ZH501 infection. Interestingly, animals sampled at 84 hpi did not have this broad cytokine response and animals sampled at 96 hpi had normal cytokine levels. These data suggest that there might be a critical transition period for RVFV pathogenesis that occured around 72 hpi, although exactly what this transition entails or the associated mechanism is unclear.

Despite a slight cytokine response in the brain following MP-12 infection, which could indicate initiation of cellular recruitment, correlating pathology was not seen. The results from MP-12 infected animals were in striking contrast to the response seen in ZH501 infected mice. A very significant pro-inflammatory cytokine response was stimulated in the brains of ZH501 infected mice and it was maintained through the course of disease. Despite the pronounced cytokine and chemokine response in the brain beginning around 48 hpi, we did not find viral antigen or histological evidence of meningoencephalitis as was observed in BALB/c mice [35]. It is surprising that we could not detect viral antigen in brain sections, as viral titers reached 5 logs in the brain when evaluated via plaque assay. Since animals were not perfused prior to tissue harvest, infectious virions could originate from residual blood in the brains. Smith *et al.* [35] did not detect meningoencephalitis until day 8 post infection while in our study none of the ZH501 infected animals survived beyond 4 dpi, in part by experimental design. Based on the Smith *et al.* study and observations during vaccine trials, it is likely that the C57BL/6 mice would have developed neurological disease had they survived longer.

Previously published studies have shown that the NSs protein of wild-type RVFV has type I IFN antagonist properties [34], while the MP-12 vaccine strain induces type I IFN in in-bred mouse strains [30], [31], [62]. Here we found that wild-type ZH501 induced a modest IFN-β response in the liver and spleen while the IFN-β response in MP-12 infected animals was negligible. The induction of the IFN-β response was not evident until 72 hpi suggesting that production of measurable IFN-β could be correlated to increases in virus titer and is similar to results reported by van Vuren in BALB/c mice [33]. Previous work has shown that the initial inhibition of type I IFN occurs shortly after infection allowing the virus to become established with the resulting release of IFN-β coming from bystander cells [30], [31]. These data are similar to results from Bouloy et al who found that acid stable IFN-α/β levels were negligible in the serum of wild-type (129/SvPasIco) mice infected with either wild-type ZH548 virus or the MP-12 virus [31]. The lack of a strong generalized immune response suggests a key localized role for IFN-β at the very onset of viral infection of macrophages or dendritic cells which could limit virus propagation and dissemination, subsequently eliminating the induction of a broad response. Evidence of virus in the spleen of some MP-12 infected animals and the moderate Th-2 type response in the brain suggests that some virus escapes initial control measures, but that the particle numbers are insufficient to trouble a competent immune system.

In this study, we have clearly identified significant host response differences in the mouse model following infection with either wild-type RVFV or the attenuated vaccine strain. We have also identified a temporal correlation between increased virus titers in ZH501 infected mice, a decrease in lymphocytes, monocytes and platelets, architectural disruption and necrosis in the liver, and the onset of a significant systemic cytokine response. The specific mechanisms driving these physiological changes still need to be elucidated. However, many of the responses that we identified can be directly correlated to disease outcome. Clearly, there is a significant amount of cellular infiltration into and necrosis of the liver that likely limits organ function, disrupts regulation of the acute phase response and the ability of the coagulation cascade to limit hemorrhaging, regardless of the mechanism associated with vascular leakage. There is also evidence that neurologic disease may play a role in the demise of ZH501 infected animals despite a lack of gross signs of neurologic disease. The finding of neurologic involvement is not surprising as we have observed a number of studies with vaccines and attenuated viruses where animals that survive the first phase of disease following ZH501 infection and are only partially protected may succumb to neurologic disease 10–14 dpi (M. Holbrook, unpublished observations). There have also been published reports of humans developing neurologic [63] and ocular disease [64], [65] as a result of RVFV infection.

This study has demonstrated that RVFV vaccine strain MP-12 infects and replicates in adult C57BL/6 mice, but does not cause acute disease, while the wild-type strain ZH501 is highly pathogenic and causes a significant innate immune response. Given that there are only 11 amino acid differences between the MP-12 strain and ZH548, and the high homology between ZH501 and ZH548, the differences in the host response are quite impressive. This study further validates the use of the mouse as a model for RVFV infection as clinical and immune response parameters can be correlated between the mouse and human disease. These studies also support the use of MP-12 as a safe and effective vaccine and on-going clinical trials will hopefully provide additional incentive for the use of MP-12 as a vaccine for RVFV.

## Supporting Information

Figure S1 **Serum cytokines.** The concentration of key cytokines in the serum of mice after mock infection or infection with MP-12 or ZH501. Shown here are the changes in actual concentration [pg/ml] of individual cytokines. Columns marked with a (#) indicate a significant change between MP-12 and mock infected mice. Columns marked with an (*) indicate a significant change between ZH501 infected and mock infected mice, while columns marked with a (+) indicate a significant difference between MP-12 and ZH501 infected animals. The numbers are the average of 5 mice ± the standard deviation between the mice except 96 hours post ZH501 infection, where only 3 surviving mice are represented.
(TIF)

Figure S2 **Liver cytokines.** The concentration of key cytokines in the liver of mice after mock infection or infection with MP-12 or ZH501. Shown here are the changes in actual concentration [pg/ml] of individual cytokines. Columns marked with a (#) indicate a significant change between MP-12 and mock infected mice. Columns marked with an (*) indicate a significant change between ZH501 infected and mock infected mice, while columns marked with a (+) indicate a significant difference between MP-12 and ZH501 infected animals. The numbers are the average of 5 mice ± the standard deviation between the mice except 96 hours post ZH501 infection, where only 3 surviving mice are represented.
(TIF)

Figure S3 **Spleen cytokines.** The concentration of key cytokines in the spleen of mice after mock infection or infection with MP-12 or ZH501. Shown here are the changes in actual concentration [pg/ml] of individual cytokines. Columns marked with a (#) indicate a significant change between MP-12 and mock infected mice. Columns marked with an (*) indicate a significant change between ZH501 infected and mock infected mice, while columns marked with a (+) indicate a significant difference between MP-12 and ZH501 infected animals. The numbers are the average of 5 mice ± the standard deviation between the mice except 96 hours post ZH501 infection, where only 3 surviving mice are represented.
(TIF)

Figure S4 **Brain cytokines.** The concentration of key cytokines in the brain of mice after mock infection or infection with MP-12 or ZH501. Shown here are the changes in actual concentration [pg/ml] of individual cytokines. Columns marked with a (#) indicate a significant change between MP-12 and mock infected mice. Columns marked with an (*) indicate a significant change between ZH501 infected and mock infected mice, while columns marked with a (+) indicate a significant difference between MP-12 and ZH501 infected animals. The numbers are the average of 5 mice ± the standard deviation between the mice except 96 hours post ZH501 infection, where only 3 surviving mice are represented.
(TIF)

Table S1 **Complete blood cell counts.** Total white blood cell concentration (WBC), lymphocyte concentration (LY), monocyte concentration (MO), eosinophil concentration (EO), neutrophil concentration (NE), total red blood cell concentration (RBD) and platelet concentration (PLT) after mock, MP-12 or ZH501 infection. Each value is the average of 5 mice with the standard deviation (SD) below, except 96 hpi in the ZH501 infected group which represents the average of the three surviving mice. K/µl = 103 cells/µl; M/µl = 106 cells/µl. *Note: n = 5 for all time points except 96 hours post ZH501 infection where n = 3. A subset of these data are presented in Figure 3.
(DOCX)
