# Supplementary figures and images for: Chemotactic and Inflammatory Responses in the Liver and Brain Are Associated with Pathogenesis of Rift Valley Fever Virus Infection in the Mouse
Source: PLoS Negl Trop Dis. 2012 Feb 28;6(2):e1529. doi: 10.1371/journal.pntd.0001529 (PMC3289610; doi:10.1371/journal.pntd.0001529)

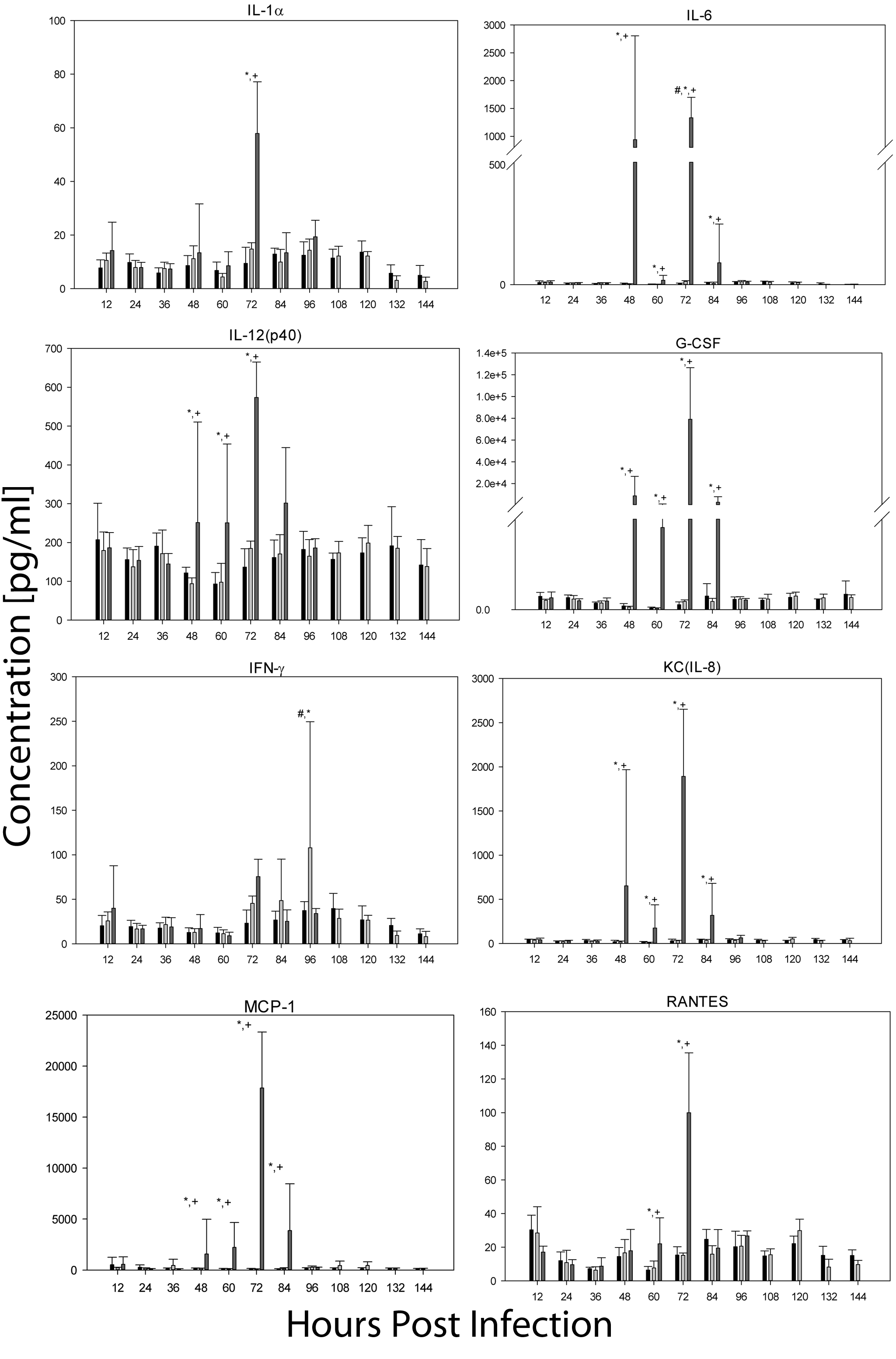

Supplement: Figure S1 — Serum cytokines. The concentration of key cytokines in the serum of mice after mock infection or infection with MP-12 or ZH501. Shown here are the changes in actual concentration [pg/ml] of individual cytokines. Columns marked with a (#) indicate a significant change between MP-12 and mock infected mice. Columns marked with an (*) indicate a significant change between ZH501 infected and mock infected mice, while columns marked with a (+) indicate a significant difference between MP-12 and ZH501 infected animals. The numbers are the average of 5 mice ± the standard deviation between the mice except 96 hours post ZH501 infection, where only 3 surviving mice are represented. (TIF) [file pntd.0001529.s001.tif]

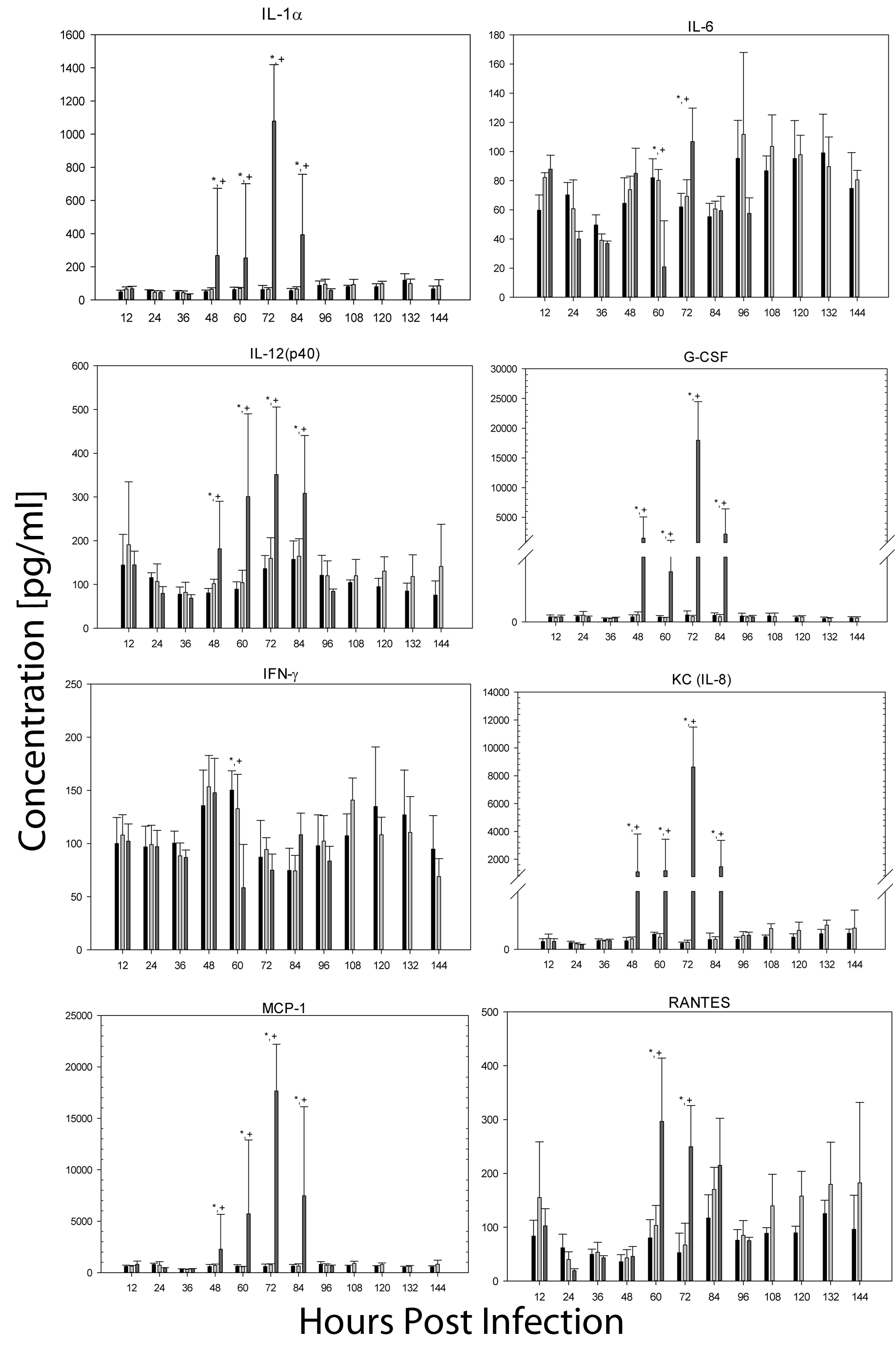

Supplement: Figure S2 — Liver cytokines. The concentration of key cytokines in the liver of mice after mock infection or infection with MP-12 or ZH501. Shown here are the changes in actual concentration [pg/ml] of individual cytokines. Columns marked with a (#) indicate a significant change between MP-12 and mock infected mice. Columns marked with an (*) indicate a significant change between ZH501 infected and mock infected mice, while columns marked with a (+) indicate a significant difference between MP-12 and ZH501 infected animals. The numbers are the average of 5 mice ± the standard deviation between the mice except 96 hours post ZH501 infection, where only 3 surviving mice are represented. (TIF) [file pntd.0001529.s002.tif]

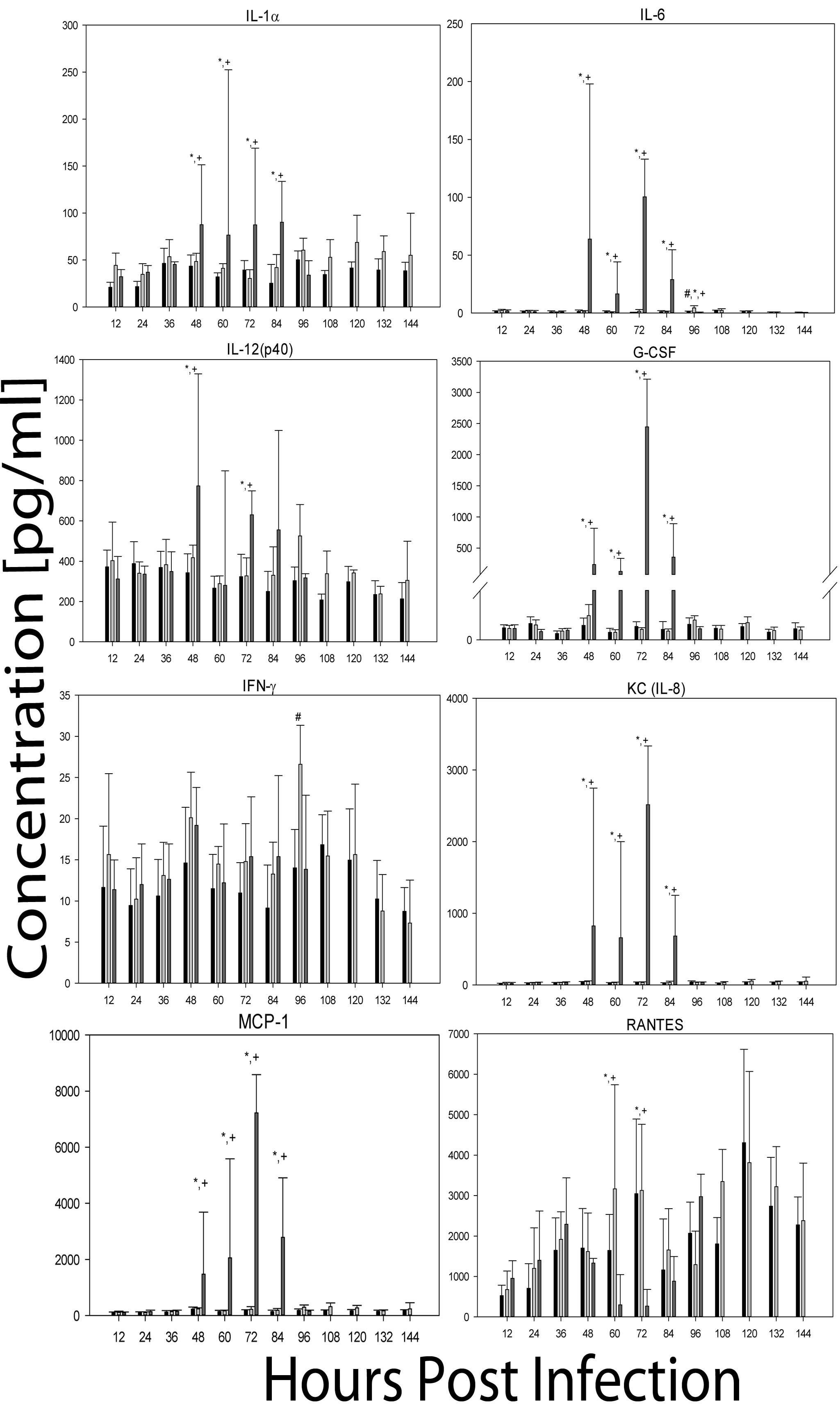

Supplement: Figure S3 — Spleen cytokines. The concentration of key cytokines in the spleen of mice after mock infection or infection with MP-12 or ZH501. Shown here are the changes in actual concentration [pg/ml] of individual cytokines. Columns marked with a (#) indicate a significant change between MP-12 and mock infected mice. Columns marked with an (*) indicate a significant change between ZH501 infected and mock infected mice, while columns marked with a (+) indicate a significant difference between MP-12 and ZH501 infected animals. The numbers are the average of 5 mice ± the standard deviation between the mice except 96 hours post ZH501 infection, where only 3 surviving mice are represented. (TIF) [file pntd.0001529.s003.tif]

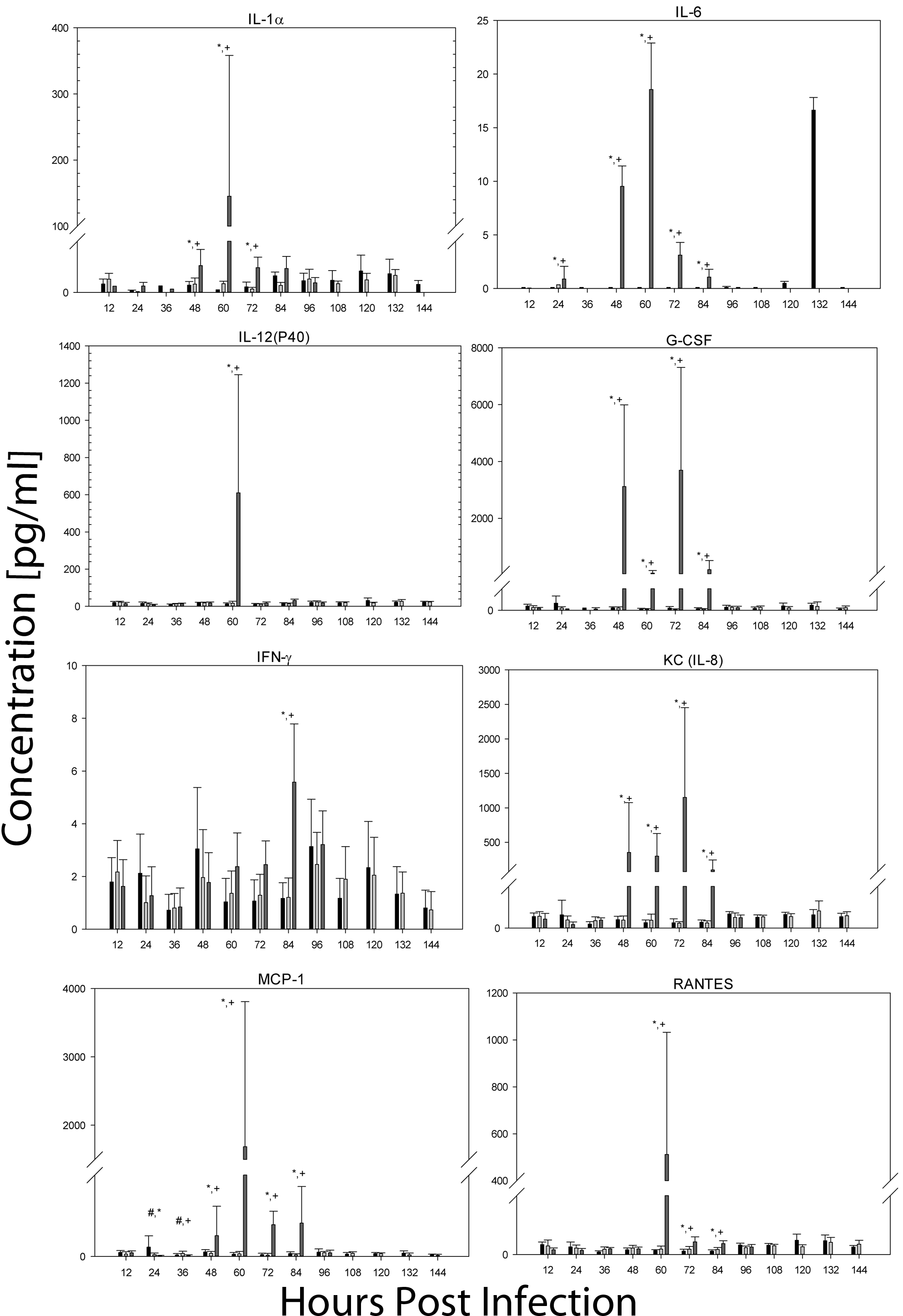

Supplement: Figure S4 — Brain cytokines. The concentration of key cytokines in the brain of mice after mock infection or infection with MP-12 or ZH501. Shown here are the changes in actual concentration [pg/ml] of individual cytokines. Columns marked with a (#) indicate a significant change between MP-12 and mock infected mice. Columns marked with an (*) indicate a significant change between ZH501 infected and mock infected mice, while columns marked with a (+) indicate a significant difference between MP-12 and ZH501 infected animals. The numbers are the average of 5 mice ± the standard deviation between the mice except 96 hours post ZH501 infection, where only 3 surviving mice are represented. (TIF) [file pntd.0001529.s004.tif]
