# Supplementary material for: Chemotactic and Inflammatory Responses in the Liver and Brain Are Associated with Pathogenesis of Rift Valley Fever Virus Infection in the Mouse
Source: PLoS Negl Trop Dis. 2012 Feb 28;6(2):e1529. doi: 10.1371/journal.pntd.0001529 (PMC3289610; doi:10.1371/journal.pntd.0001529)
Supplement: Table S1 — Complete blood cell counts. Total white blood cell concentration (WBC), lymphocyte concentration (LY), monocyte concentration (MO), eosinophil concentration (EO), neutrophil concentration (NE), total red blood cell concentration (RBD) and platelet concentration (PLT) after mock, MP-12 or ZH501 infection. Each value is the average of 5 mice with the standard deviation (SD) below, except 96 hpi in the ZH501 infected group which represents the average of the three surviving mice. K/µl = 103 cells/µl; M/µl = 106 cells/µl. *Note: n = 5 for all time points except 96 hours post ZH501 infection where n = 3. A subset of these data are presented in Figure 3. (DOCX) [file pntd.0001529.s005.docx]

|  | Normal | Hours Post Infection | | | | | | | | | | | | |
| --- | --- | --- | --- | --- | --- | --- | --- | --- | --- | --- | --- | --- | --- | --- |
| Parameter | Values | 0 | 12 | 24 | 36 | 48 | 60 | 72 | 84 | 96 | 108 | 120 | 132 | 144 |
| **Mock (N=5)** |  |  |  |  |  |  |  |  |  |  |  |  |  |  |
| WBC (K/μl) | 1.8 - 10.7 | 5.89 (1.5) | 2.16 (0.5) | 2.93 (0.9) | 3.88 (2.1) | 2.00 (0.7) | 1.55 (0.7) | 6.90 (2.4) | 2.84 (1.7) | 4.22 (1.9) | 1.54 (0.8) | 3.72 (2.3) | 4.41 (1.5) | 5.20 (1.1) |
| LY (K/μl) | 0.9 - 9.3 | 4.62 (1.2) | 2.21 (1.2) | 2.08 (0.6) | 2.95 (1.5) | 1.28 (0.5) | 1.24 (0.6) | 5.04 (1.6) | 2.16 (1.4) | 3.13 (1.4) | 1.15 (0.6) | 2.74 (1.6) | 3.60 (1.4) | 3.44 (1.1) |
| MO (K/μl) | 0.0 - 0.4 | 0.22 (0.07) | 0.14 (0.02) | 0.15 (0.1) | 0.25 (0.2) | 0.1 (0.07) | 0.07 (0.02) | 0.47 (0.4) | 0.17 (0.1) | 0.22 (0.1) | 0.10 (0.07) | 0.21 (0.2) | 0.14 (0.02) | 0.57 (0.4) |
| EO (K/μl) | 0.0 - 0.2 | 0.04 (0.03) | 0.03 (0.04) | 0.06 (0.1) | 0.08 (0.07) | 0.07 (0.06) | 0.01 (0.01) | 0.11 (0.04) | 0.07 (0.06) | 0.03 (0.02) | 0.04 (0.03) | 0.04 (0.04) | 0.02 (0.01) | 0.11 (0.05) |
| NE (K/μl) | 0.1 - 2.4 | 0.99 (0.4) | 0.42 (0.1) | 0.64 (0.3) | 0.58 (0.4) | 0.55 (0.1) | 0.23 (0.1) | 1.25 (0.5) | 0.43 (0.3) | 0.83 (0.4) | 0.24 (0.1) | 0.72 (0.6) | 0.67 (0.1) | 1.06 (0.3) |
| RBC (M/μl) | 6.36 - 9.42 | 9.47 (0.3) | 9.22 (1.0) | 8.76 (2.2) | 9.39 (0.2) | 9.95 (0.2) | 9.62 (0.3) | 10.3 (1.7) | 8.97 (0.5) | 10.43 (0.4) | 9.92 (0.3) | 8.99 (0.6) | 9.55 (0.2) | 8.79 (1.4) |
| PLT (K/μl) | 592 - 2972 | 291.33 (34.56) | 460.50 (392.85) | 488.75 (216.64) | 618.00 (105.82) | 564.75 (109.43) | 529.60 (129.85) | 452.25 (200.82) | 455.20 (301.84) | 408.60 (178.0) | 716.20 (87.15) | 484.20 (221.24) | 719.00 (107.75) | 434.20 (371.66) |
| **MP-12 (N=5)** |  |  |  |  |  |  |  |  |  |  |  |  |  |  |
| WBC (K/μl) |  |  | 5.01 (1.6) | 2.70 (1.3) | 2.70 (1.3) | 5.19 (0.9) | 3.52 (1.4) | 4.33 (0.7) | 4.72 (1.6) | 3.45 (1.3) | 2.00 (0.7) | 4.66 (1.5) | 5.10 (5.5) | 3.27 (1.4) |
| LY (K/μl) |  |  | 3.41 (1.2) | 2.12 (1.1) | 3.57 (0.5) | 3.57 (0.5) | 2.93 (1.3) | 3.22 (0.7) | 3.73 (1.1) | 2.53 (1.2) | 1.32 (0.5) | 3.21 (1.3) | 3.93 (4.9) | 2.40 (1.3) |
| MO (K/μl) |  |  | 0.15 (0.13) | 0.37 (0.21) | 0.33 (0.3) | 0.21 (0.04) | 0.13 (0.04) | 0.13 (0.04) | 0.16 (0.06) | 0.26 (0.1) | 0.25 (0.1) | 0.26 (0.1) | 0.30 (0.2) | 0.18 (0.1) |
| EO (K/μl) |  |  | 0.04 (0.07) | 0.02 (0.07) | 0.02 (0.03) | 0.09 (0.07) | 0.04 (0.05) | 0.02 (0.01) | 0.08 (0.04) | 0.03 (0.04) | 0.08 (0.07) | 0.03 (0.02) | 0.04 (0.03) | 0.02 (0.03) |
| NE (K/μl) |  |  | 0.43 (0.4) | 1.03 (0.2) | 0.42 (0.2) | 1.12 (0.4) | 0.47 (0.1) | 0.93 (0.3) | 0.66 (0.5) | 0.63 (0.2) | 0.43 (0.2) | 1.14 (0.3) | 0.82 (0.6) | 0.65 (0.1) |
| RBC (M/μl) |  |  | 9.24 (0.65) | 13.48 (1.8) | 8.94 (1.23) | 8.05 (1.4) | 10.13 (0.3) | 9.50 (0.5) | 9.07 (0.3) | 8.50 (2.2) | 10.21 (0.4) | 9.76 (0.4) | 11.76 (4.7) | 8.41 (1.3) |
| PLT (K/μl) |  |  | 539.50 (164.1) | 509.00 (215.0) | 652.80 (170.3) | 542.67 (37.3) | 395.20 (129.5) | 530.20 (76.4) | 570.00 (55.8) | 449.50 (238.4) | 746.40 (50.82) | 574.00 (100.4) | 644.00 (178.3) | 681.3 (121.9) |
| **ZH501 (N=5)*** |  |  |  |  |  |  |  |  |  |  |  |  |  |  |
| WBC (K/μl) |  |  | 1.95 (1.6) | 4.62 (1.8) | 3.29 (2.5) | 4.16 (2.5) | 1.07 (0.4) | 1.42 (0.1) | 2.55 (1.3) | 4.47 (0.2) |  |  |  |  |
| LY (K/μl) |  |  | 1.33 (0.9) | 3.50 (1.7) | 2.59 (1.9) | 2.70 (1.8) | 0.56 (0.4) | 0.34 (0.03) | 1.62 (1.4) | 3.04 (0.6) |  |  |  |  |
| MO (K/μl) |  |  | 0.15 (0.1) | 0.19 (0.07) | 0.18 (0.16) | 0.18 (0.1) | 0.06 (0.04) | 0.02 (0.03) | 0.18 (0.1) | 0.60 (0.3) |  |  |  |  |
| EO (K/μl) |  |  | 0.04 (0.07) | 0.05 (0.04) | 0.05 (0.05) | 0.09 (0.07) | 0.01 (0.02) | 0.02 (0.01) | 0.06 (0.03) | 0.15 (0.04) |  |  |  |  |
| NE (K/μl) |  |  | 0.43 (0.4) | 0.86 (0.2) | 0.46 (0.3) | 1.18 (0.7) | 0.44 (0.2) | 1.04 (0.1) | 0.69 (0.3) | 0.64 (0.3) |  |  |  |  |
| RBC (M/μl) |  |  | 9.24 (0.7) | 9.21 (0.2) | 9.41 (0.5) | 9.22 (0.5) | 9.31 (0.7) | 6.39 (1.33) | 8.95 (1.1) | 8.59 (0.6) |  |  |  |  |
| PLT (K/μl) |  |  | 539.50 (164.1) | 584.40 (132.0) | 672.75 (37.0 | 440.20 (183.0) | 563.20 (239.4) | 112.00 (43.8) | 383.80 (109.8) | 174.00 (165.8) |  |  |  |  |
